# Preparation of Salt-Induced Ultra-Stretchable Nanocellulose Composite Hydrogel for Self-Powered Sensors

**DOI:** 10.3390/nano13010157

**Published:** 2022-12-29

**Authors:** Xiaofa Wang, Xincai Li, Baobin Wang, Jiachuan Chen, Lei Zhang, Kai Zhang, Ming He, Yu Xue, Guihua Yang

**Affiliations:** 1 State Key Laboratory of Biobased Material and Green Papermaking, Qilu University of Technology, Shandong Academy of Sciences, Jinan 250353, China; 2 Guangxi Key Laboratory of Clean Pulp & Papermaking and Pollution Control, College of Light Industry and Food Engineering, Guangxi University, Nanning 530004, China

**Keywords:** cellulose nanocrystals (CNCs), hydrogel, self-powered sensor, triboelectric nanogenerator (TENG), strain sensor

## Abstract

Hydrogels have attracted much attraction for promising flexible electronics due to the versatile tunability of the properties. However, there is still a big obstacle to balance between the multi-properties and performance of wearable electronics. Herein, we propose a salt-percolated nanocellulose composite hydrogel which was fabricated via radical polymerization with acrylic acid as polymer networks (NaCl-CNCs-PAA). CNCs were utilized as a reinforcing agent to enhance the mechanical properties of the hydrogel. Moreover, the abundant hydroxyl groups endow the hydrogel with noncovalent interactions, such as hydrogen bonding, and the robustness of the hydrogel was thus improved. NaCl incorporation induced the electrostatic interaction between CNCs and PAA polymer blocks, thus facilitating the improvement of the stretchability of the hydrogel. The as-obtained hydrogel exhibited excellent stretchability, ionic conductivity, mechanical robustness and anti-freezing properties, making it suitable for self-powered sensing applications. A single-mode triboelectric nanogenerator (C-TENG) was fabricated by utilizing the composite hydrogel as electrodes. This C-TENG could effectively convert biomechanical energy to electricity (89.2 V, 1.8 µA, 32.1 nC, and the max power density of 60.8 mW m^−2^ at 1.5 Hz.) Moreover, the composite hydrogel was applied for strain sensing to detect human motions. The nanocellulose composite hydrogel can achieve the application as a power supply in integrated sensing systems and as a strain sensor for human motion detection.

## 1. Introduction

The booming development of artificial intelligence and Internet of Things (IoT) inspires the next generation of wearable electronics [1,2,3]. The conventional passive sensors integrated with power supply suffer in regard to portability and long-lasting utilization, as they hardly meet the requirements for the current wearable devices. Triboelectric nanogenerators derived flexible self-powered sensors which can fulfill the power supply function, as well as sensing applications, demonstrate a promising candidate for the next generation of sensors [4,5,6].

Hydrogels are hydrophilic polymer networks infiltrated with abundant water/ionic liquid [7,8]. The highly tunable properties, such as mechanical property, ionic conductivity, and biocompatibility, enabled the application of hydrogels as the electrode part of TENGs. The common single-network hydrogels are restricted by the inferior mechanical robustness and durability [9,10]. Nanocomposite incorporation is considered to be an efficient strategy to promote the mechanical properties of the hydrogel [11,12]. By introducing noncovalent interactions into the hydrogel matrix [13,14], the mechanical properties, such as mechanical robustness, stretchability, and durability, could thus be enhanced. Moreover, other properties, such as anti-freezing and conductivity, are essential for the utilization of TENG applied under severe conditions. Salt insertion into the polymer networks is another facile and low-cost strategy to achieve excellent ionic conductivity and anti-freezing properties, as is favorable for the practical application of wearable devices working under extreme environmental conditions [15,16,17,18].

Due to the renewable and abundant-source nature of cellulose [19], cellulose nanocrystals (CNCs) have attracted much attention due to their unique properties, such as excellent mechanical properties, reinforcing capability, low density, and biodegradability [20,21]. Moreover, CNCs acting as nanofillers could be utilized as dispersing agents and reinforcing agents to enhance the toughness and stretchability of functional hydrogels [22,23,24,25]. Thanks to the functional groups on the side chain of CNCs, noncovalent interactions including hydrogen bonding and electrostatic interactions would be introduced with CNCs’ incorporation, which would act as a sacrificial bond for promoting the stretchability of hydrogels. Yang et al. reported that the transition of the isolation form of CNCs under dilute concentration to the spatially continuous form over critical concentration enhanced the mechanical properties of a CNCs–PAA hydrogel [26]. Aref et al. reported that the incorporation of NaCl into the CNCs–PVA hydrogel system induced the CNCs’ aggregation, which facilitated the enhancement of the mechanical properties of the hydrogel [27].

Herein, a CNCs-incorporated composite hydrogel was fabricated via radical polymerization, using polyacrylic as the building blocks, along with the insertion of NaCl. The CNCs acted as dispersing and reinforcing reagents to improve the mechanical properties of the hydrogel. Moreover, NaCl was inserted to the hydrogel matrix to enhance the conductivity and anti-freezing properties which would favor its application as a self-powered device in extreme conditions. Notably, the synergistic effect of CNCs and NaCl endowed the hydrogel with multifunctionalities, including ionic conductivity, stretchability, toughness and anti-freezing properties. The multifunctional hydrogel was integrated with VHB tapes for utilization as triboelectric nanogenerator and strain sensors. The self-powered device demonstrated promising applications in human motion and bioelectrical signal detection.

## 2. Materials and Methods

### 2.1. Materials

Cellulose nanocrystals (CNCs) were kindly provided by Cellulose Lab Corp, NB, Canada. N,N′-Methylenebis (acrylic) (MBA) and ammonium persulfate (APS) were purchased from Shanghai Aladdin Biochemical Technology Co., Ltd., Shanghai, China. Acrylic acid, sodium chloride (NaCl), and N,N,N’,N’-Tetramethylethylenediamine (TMEDA) were purchased from Shanghai Maclean Biochemical Technology Co., Shanghai, China. NaCl was purchased from Sinopharm Chemical Reagent Co., Ltd., Shanghai, China. All chemicals were of reagent grade, without any further purification. All experiments were conducted using deionized water.

### 2.2. Preparation of CNCs Composite Hydrogel

A total of 0.5 g of CNCs’ powder was added into a beaker containing 99.5 g of water, and 0.5 wt.% CNCs suspension was mixed evenly with an emulsifying machine for 20 min. The hydrogel was prepared in two steps. In the first step, 5 mL of deionized water was added to a different amount of CNC dispersion (0.5 wt.%). A different amount of NaCl was added to the CNC suspension, and it was fully dissolved by mechanical stirring under 700 rpm. Then 2.5 mL of acrylic acid was added, with magnetic stirring at 700 rpm for 10 min, at room temperature. In the second step, 1.25 mg of N,N′-Methylenebis(acrylic) (MBA) and 12.5 mg of ammonium persulfate were mixed into the previous solution, and the solution was then purged with N_2_ for 2 min to remove air. Finally, 10 μL of N,N,N′,N′-Tetramethyl ethylenediamine (TMEDA) was added, with magnetic stirring at 700 rpm for 5 min, at room temperature. Then the solution was poured into a 60 mm plastic Petri dish and placed into a 60 °C blast-drying oven for 90 min. The sample was denoted as PC_x_N_y_. The x and y represented the amount of CNC and NaCl, respectively.

### 2.3. Assembly of Strain Sensor

The as-prepared hydrogel was used as a conductive electrode, and two silver wires were connected to both ends of the hydrogel. Then it was composited with VHB films to inhibit dewatering; the hydrogel-based strain sensor was then attached to the volunteer joints to detect the sensing signals with different motions.

### 2.4. Assembly of Triboelectric Nanogenerators (TENG)

The as-prepared hydrogel (3 cm × 3.5 cm × 4 mm) was composited with two VHB films (3 M, 3 cm × 3.5 cm × 5 mm), and a copper wire was attached to one side of the hydrogel to serve as a current collector. The triboelectric nanogenerator was utilized for measuring specific properties.

### 2.5. Characterizations

A linear motor was used to provide the input of mechanical motions. For all the tests of energy generation of the C-TENG, the pressure (100 kPa), speed (0.3 m/s), and frequency (~1.5 Hz) of the step motor were fixed. The voltage, current, and charge quantity were recorded by a Keithley electrometer (6514). The mechanical tensile test and stretch cycling test of the STENGs were conducted by an TA.XT PlusC/Texture Analyzer. For the tensile test, the strain rate was fixed at 30 mm/min. For the tension compression cycle, the strain rate was fixed at 180 mm/min. For the anti-dehydration test, PAA–CNC_0.25%_ and PAA–CNC_0.25%_–NaCl_5.5_ hydrogels at room temperature were used; they were weighed every 24 h, and we took the average value of the three groups. All the data were tested in parallel three groups of the experiment. The real-time electrical signals of the strain sensors based on the resistance changes of the hydrogels in different states were recorded by a TongHui TH2832 LCR digital bridge, Shanghai, China. ECG and EMG were recorded in real time by an RM6240EC multichannel physiological signal acquisition and processing system, Chengdu, China. Fourier-transform infrared (FTIR) spectroscopy of the samples was recorded by using an Alpha infrared spectrometer (Thermofisher, Waltham, MA, USA) at room temperature. The fracture cross-sections of the hydrogel were observed by scanning electron microscope (SEM, Hitachi Regulus 8220, Tokyo, Japan). After being freeze-dried for 48 h, the hydrogel samples were sprayed with gold for 5 min at the accelerated voltage of 5 kV.

## 3. Results and Discussion

### 3.1. Schematic of the Nanocellulose Composite Hydrogel for Self-Powered Devices

**Figure 1:**
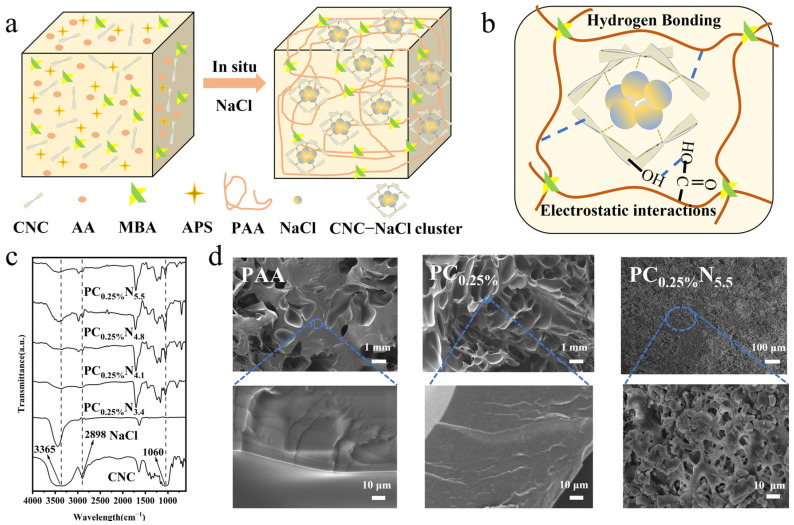
Preparation of salt-percolated nanocellulose composite hydrogel. (**a**) In situ polymerization of the nanocellulose composite hydrogel. (**b**) Possible noncovalent interactions within the hydrogel matrix. (**c**) FTIR of the CNC and nanocellulose composite hydrogel. (**d**) SEM of the PAA, PC_0.25%_, and PC_0.25%_N_5.5_ hydrogel under different magnifications.

Wearable self-powered sensors have raised a great challenge for the current functional materials with respect to conductivity, mechanical properties, anti-freezing properties, etc. Conventional hydrogels as ionic conductors are plagued by inferior properties, including their conductivity, toughness, and stretchability. To deal with these issues, a salt-percolated nanocellulose composite hydrogel was fabricated via radical polymerization with acrylic as polymer networks (NaCl–CNCs–PAA) (Figure 1a). CNCs were utilized as a reinforcing agent to enhance the mechanical properties of the hydrogel. Moreover, the abundant hydroxyl groups endowed the hydrogel with noncovalent interactions, such as hydrogen bonding, and the robustness of the hydrogel was thus improved (Figure 1b). The incorporation of NaCl induced the aggregation of CNCs in the hydrogel matrix due to the ionic interactions with NaCl, and the noncovalent bonding interactions were thus enhanced within CNCs–CNCs and CNCs–PAA polymer blocks, which act as sacrificial bonds to facilitate the improvement of stretchability of the hydrogel [27,28]. The as-obtained hydrogel exhibited excellent stretchability, ionic conductivity, mechanical robustness, and anti-freezing properties, which are favorable for the application of self-powered sensors in diverse environments.

FTIR was used to investigate the chemical structure of the hydrogel (Figure 1c); the spectrum of CNCs showed peaks at 3365 cm^−1^ and 2898 cm^−1^, which are assigned to the OH stretching and CH_2_ stretching vibrations. Moreover, the PAA polymer matrix showed that the absorptions at 1558, 1450, and 1647 cm^−1^ were assigned to the asymmetrical and symmetrical stretching vibration of COO– and the bond stretching vibrations of C–C [29]. After the incorporation of NaCl, the stretching vibration peak of –OH and –COO– was shifted, implying the interactions within the hydrogel matrix among poly acrylic acid, CNCs, and NaCl. SEM was utilized to the verify the morphology of the hydrogel. As shown in Figure 1d, the PC_0.25%_ hydrogel exhibited a more porous structure than the PAA hydrogel, and the little dots on the hydrogel matrix may be related to the incorporation of CNCs. Furthermore, the average pore diameter of the PC_0.25%_N_5.5_ hydrogel decreased dominantly compared with that of the PC_0.25%_ hydrogel, and there were round-shaped aggregations within the hydrogel matrix. This may be attributed to the CNCs–NaCl aggregation induced by electrostatic interactions.

### 3.2. Properties of the Nanocellulose Composite Hydrogel

**Figure 2:**
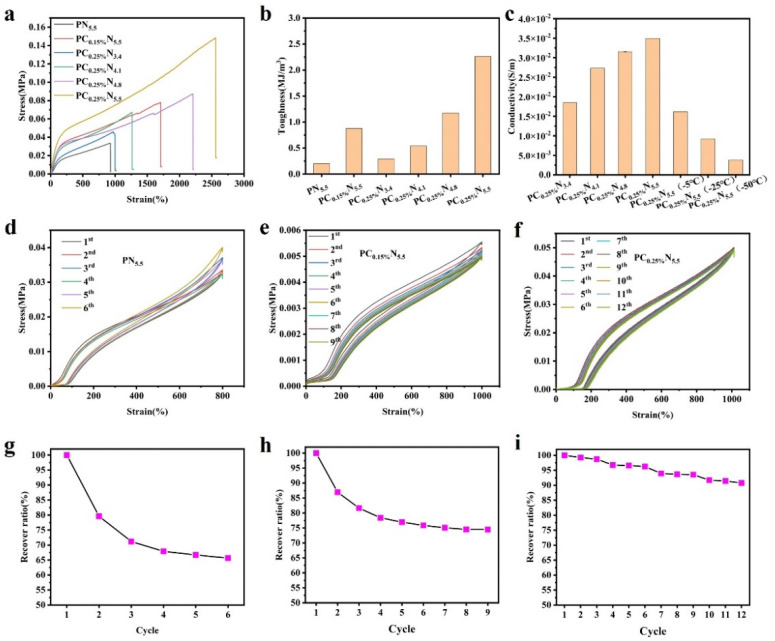
Properties of the nanocellulose composite hydrogel. (**a**) Tensile strain curves of different hydrogels. (**b**) Toughness of different hydrogels. (**c**) Ionic conductivity of different hydrogels. (**d**) Repetitive loadingunloading stress—strain curve of the PN_5.5_ hydrogel for 6 cycles (the inset shows the corresponding recovery ratio for each cycle). (**e**) Repetitive loading—unloading stress—strain curve of the PC_0.15%_N_5.5_ hydrogel for 9 cycles (the inset shows the corresponding recovery ratio for each cycle). (**f**) Repetitive loading—unloading stress—strain curve of the PC_0.25%_N_5.5_ hydrogel for 12 cycles (the inset shows the corresponding recovery ratio for each cycle). (**g**) The PN_5.5_ hydrogel repeated loading—unloading stress—strain recovery ratio. (**h**) The PC_0.15%_N_5.5_ hydrogel repeated loading—unloading stress—strain recovery ratio. (**i**) The PC_0.25%_N_5.5_ hydrogel repeated loading—unloading stress—strain recovery ratio.

The properties of the composite hydrogel were determined with respect to the stress, toughness, ionic conductivity, and durability. Figure 2a exhibits the stress–strain curve of the composite hydrogel with varying CNC and NaCl contents. The results showed that the mechanical strength of the hydrogel was enhanced with the increase of the CNC addition, and the elasticity of the nanocomposite hydrogel was also enhanced with the increase of the CNC and NaCl content in the hydrogel. The insertion of NaCl with a higher concentration led to higher ionic strength, which result in an enhanced aggregation effect for the CNCs. Thus, the mechanical properties were facilitated/induced by the energy-dissipation mechanism. It is noteworthy that the synergistic effect of the CNCs and NaCl could endow the hydrogel with excellent stretchability (2600%). Yang et al. reported that the CNCs’ incorporation in the PAA matrix resulted in a higher elongation rate (>1100%) than the pure PAA hydrogel [26]. The toughness was also improved by introducing CNCs and NaCl into the hydrogel; as demonstrated in Figure 2b, the toughness achieved 2.3 MJ/m^3^, which may be due to the aggregation of CNCs induced by the elevated ionic strength with the addition of NaCl [30]. Thus, the mechanical properties were enhanced with the extra physical interactions within CNCs–CNCs and CNCs–PAA.

Ionic conductivity is essential for the application of hydrogels for flexible electronics. Figure 2c displays the ionic conductivity of hydrogels with the incorporation of different NaCl concentrations. The ionic conductivity was increased from 1.8 S/cm to 3.5 S/cm with the increase of the concentration of the incorporated NaCl. The ionic conductivity of the nanocomposite hydrogel was enhanced by introducing ions which would transport freely within the hydrogel matrix. The hydrogel also showed conductivity with −50 °C, which indicated that the hydrogel could be applied under extreme conditions.

In addition, due to the introduction of the NaCl and CNCs, noncovalent interactions formed through ionic interactions and the hydrogen bonding effect, which endowed the hydrogel with excellent recovery capability for the nanocomposite hydrogel. The repeated loading–unloading cycles were performed with different hydrogels. As shown in Figure 2d, an obvious decrease of the maximum stress was found with six cycles for the NaCl-incorporated PAA hydrogel. As the addition of the CNCs increased (Figure 2e,f), the minor decrease of the residual stress (0.001 MPa) and recovery ratio (90.75%) after 12 cycles demonstrated the effect of sufficient energy dissipation induced by the nanoparticle incorporation and aggregation effect.

### 3.3. Nanocellulose Composite Hydrogel for Triboelectric Nanogenerator

**Figure 3:**
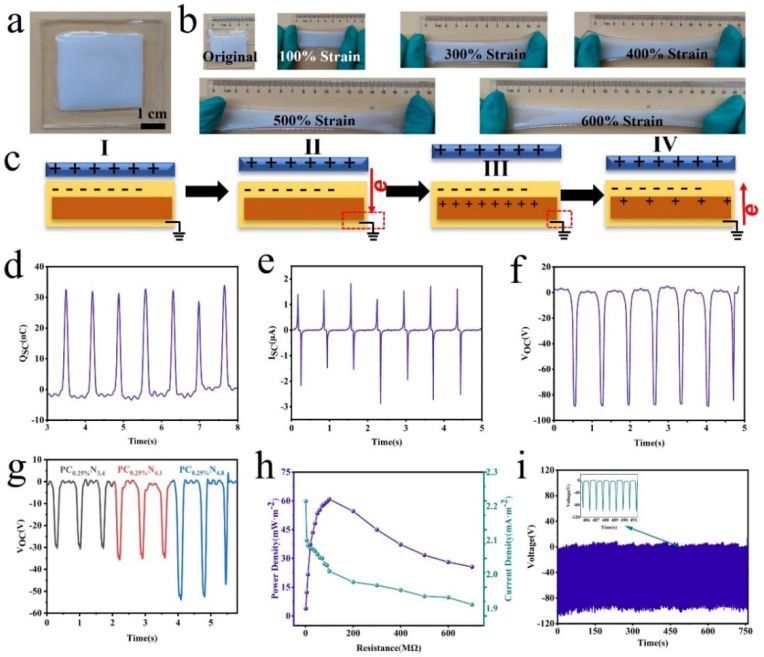
Performance of single-mode C-TENG at 1.5 Hz. (**a**) Image of the assembled C-TENG at 1.5 Hz. (**b**) Photos of C-TENG stretched under different strains at 1.5 Hz. (**c**) Mechanism of the C-TENG at 1.5 Hz. (**d**) Qsc of the C-TENG at 1.5 Hz. (**e**) Isc of the C-TENG at 1.5 Hz. (**f**) Voc of the C-TENG at 1.5 Hz. (**g**) Performance of single-mode C-TENG with different NaCl concentrations at 1.5 Hz. (**h**) Voc of C-TENG with 750 cycles at 1.5 Hz. (**i**) Power density and current density of the C-TENG at 1.5 Hz. (I–IV are stages of working mechanism).

Triboelectric nanogenerators (TENGs) are a promising strategy for utilizing low-grade mechanical energy such as human motion and convert it to electricity [31,32,33]. The ubiquitous mechanical energy recovery provided the power supply for versatile wearable electronics. The conventional electrode part of TENGs utilizes petroleum-based polymers, including polydimethylsiloxane, polyurethane, and butyl acrylate, which suffer from poor mechanical properties, such as non-renewability and poor biocompatibility. The nanocellulose composite hydrogel presented excellent mechanical properties, including stretchability, anti-freezing properties, and ionic conductivity, which make it suitable for to serve as an electrode for a TENG. The nanocellulose-composite-hydrogel-based triboelectric nanogenerator (C-TENG) with a single electrode mode was proposed. Figure 3a exhibits the schematic of the C-TENG, and the nanocellulose composite hydrogel film (3 cm × 3.5 cm × 4 mm), serving as an electrode, was embedded into two VHB tapes (4.5 cm × 5 cm ×5 mm). Due to the high stretchability of the nanocellulose composite hydrogel, the assembled C-TENG could retain its integrity after 10 cycles under 600% strain (Figure 3b). The single mode of the C-TENG works based on the effect of electrostatic induction and contact electrification, as demonstrated in Figure 3c. When the dielectric materials touched the surface of the C-TENG, the dielectric films became positively charged, and meanwhile an equivalent negative charge occurred at the surface of the C-TENG (I). Once the dielectric film moved away from the VHB surface, the ions transported to either side of the hydrogel, leading to positive ions and negative ions being aggregated at the interface of VHB-composite hydrogel and Cu-composite hydrogel, respectively. Thus, an electrical double layer formed due to the polarization effect (II). Then the excessive negative ions flowed via the Cu wire to the ground until a neutral state was achieved (III). At the final stage, the dielectric film moved back to the VHB’s surface again, and the electrons would flow from the ground to the Cu-composite hydrogel interface in the opposite direction (IV). The repetitive contact–separation cycles between the dielectric films and VHB film produced the alternating electric power [34].

The Voc, transferred charge (Qsc), and short-circuit current (Isc) of the C-TENG are shown in Figure 3d–f. It is obvious that the Voc, Qsc, and Isc showed a stable state at 89.2 V, 32.1 nC, and 1.8 uA, thus implying that the C-TENG could continuously gather the biomechanical energy and convert it to electricity to realize self-powered electronics. Moreover, the external loading of resistance is crucial for the output performance of the C-TENG. The output voltage increased from 30.5 V to 55.2 V with the increasing of the NaCl concentration from 3.4 M to 4.8 M (Figure 3g). The output voltage, current density, and power density of the C-TENG were further studied to investigate the practical performance of the C-TENG. Based on Ohm’s law, the output increased with the increase of load resistance from 1 MΩ to 700 MΩ, while the current density decreased with the increase of external resistance loading. Moreover, a maximum power density for the C-TENG reached 60.8 mW m^−2^ with the loading of resistance at 100 MΩ (Figure 3h). Furthermore, the durability of the C-TENG was investigated at 1.5 Hz for 750 cycles (Figure 3i). Thanks to the excellent mechanical properties induced by the nanocomposite and CNCs’ aggregated related noncovalent bonding, the output voltage of the C-TENG was stable, which is beneficial for practical applications.

### 3.4. Nanocellulose Composite Hydrogel for Human Motion Detection

**Figure 4:**
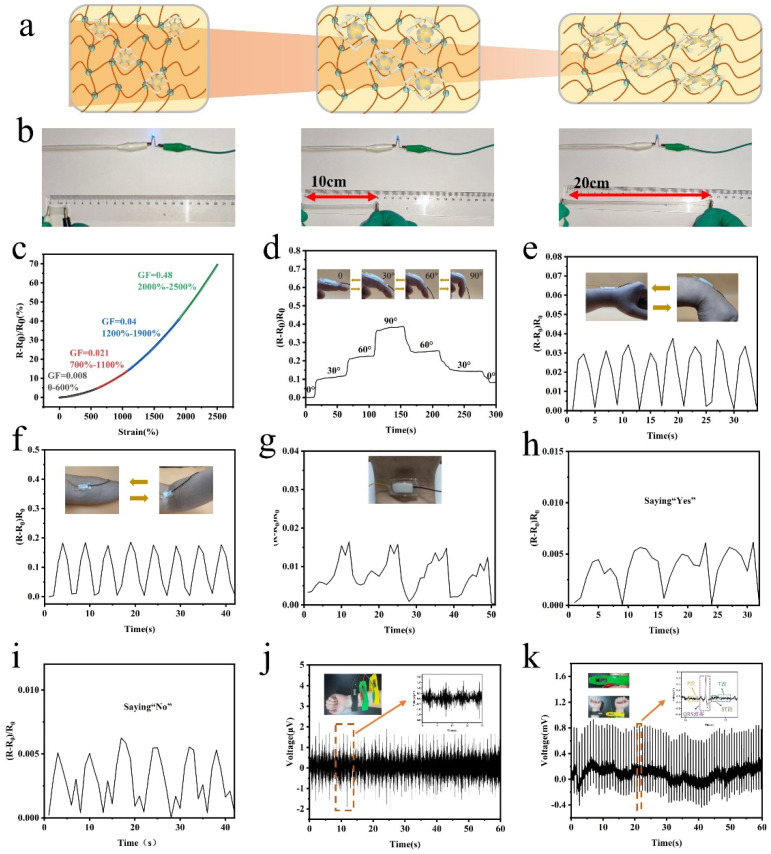
Performance of nanocellulose—composite hydrogel—based strain sensor. (**a**) Schematic of nanocellulose composite hydrogel under different strains. (**b**) Photos of the nanocellulose composite hydrogel under different strains assembled with an LED light in a circuit, indicating the LED response under different strains. (**c**) Relative resistance variation curves of the PC_0.25%_N_5.5_ hydrogel as a function of strain. (**d**) Sensing signals of finger bending under different angles (30°, 60°, and 90°). (**e**) Sensing signals of wrist bending. (**f**) Sensing signals of elbow bending. (**g**) Sensing signals of swallowing. (**h**) Sensing signals of speaking “Yes”. (**i**) Sensing signals of speaking “No”. (**j**) EMG signals detection. (**k**) ECG signals’ detection.

Due to the versatile properties of the nanocellulose composite hydrogel, the application of the novel hydrogel as a strain sensor was further studied. The ionic conductivity of the composite hydrogel was derived from the NaCl incorporation, and the mechanical deformation would induce resistance variation. The mechanism for the nanocellulose-composite-hydrogel-based strain sensor is shown in Figure 4a. The conductivity varied with the shape deformation induced by the change of ions’ transportation in the hydrogel matrix. The composite hydrogel was integrated into a circuit with a battery and LED light, and the brightness of the LED light in the circuit decreased obviously with the strain varying from 100% to 1000% (Figure 4b). The gauge factor is a critical parameter to verify the sensitivity of the strain sensor. As shown in Figure 4c, the hydrogel-based strain sensor exhibited linear sensitivity under different strain ranges, and the composite hydrogel demonstrated excellent sensitivity over a broad strain range (0–2500%). Moreover, the composite hydrogel was attached to different parts of the volunteer’s body to detect different motions. The repetitive finger bending with varied angles (30°, 60°, and 90°) could be tested with different resistance variations (0.1, 0.2, and 0.3), and the increased bending angle would induce enhanced shape deformation of the hydrogel, leading to higher resistance variation. The resistance variation stayed unchanged under the same bending angle, thus indicating the reliability of the hydrogel-based strain sensors (Figure 4d). Furthermore, the hydrogel was used for other large motion-detection applications, such as elbow and wrist bending, and the repeatable bending acquired stable resistance variation (Figure 4e,f). More interestingly, the composite hydrogel could be utilized for the detection of small motions, which verified that the composite hydrogel has excellent sensitivity. When applied on the throat of the volunteer, the sensor could differentiate between different small vibrations that occurred when there were changes in the throat of the volunteer, such as swallowing (Figure 4g), speaking “Yes” (Figure 4h), and speaking “No” (Figure 4i). The hydrogel could also be utilized as an electrode for the detection of biological signals such as EMG (Figure 4j) and ECG (Figure 4k), and it demonstrated reliable signals compared with commercial electrode materials. The composite hydrogel showed broad application as a strain sensor for the detection of human motions.

## 4. Conclusions

A salt-percolated nanocellulose composite hydrogel was prepared via radical polymerization with polyacrylic acid as polymer networks (NaCl–CNCs–PAA). CNCs were utilized as a reinforcing agent to enhance the mechanical properties of the hydrogel. The incorporation of NaCl induced the electrostatic interaction between the CNCs and PAA polymer blocks, thus facilitating the improvement of the stretchability of the hydrogel. The as-obtained hydrogel showed excellent stretchability (2574.81%), ionic conductivity (3.5 S/cm), mechanical robustness (2.26 MJ/m^3^), and anti-freezing properties (−50 °C), making it suitable for self-powered sensing applications. A single-mode triboelectric nanogenerator was fabricated by utilizing the composite hydrogel as electrodes. This C-TENG could effectively convert biomechanical energy to electricity (89.2 V, 1.8 µA, and 32.1 nC, and the max power density of 60.8 mW m^−2^ at 1.5 Hz). Due to the strain sensitivity induced by ionic conductivity, the composite hydrogel was applied for strain sensing to detect human motions, including wrist bending, finger bending, and elbow bending. The nanocellulose composite hydrogel will inspire the fabrication of novel nanocellulose-based hydrogels and its application in future wearable electronics.

## Data Availability

All the data are presented in the manuscript.
